# Patient, clinician and manager experience of the accelerated implementation of virtual consultations following COVID‐19: A qualitative study of preferences in a tertiary orthopaedic rehabilitation setting

**DOI:** 10.1111/hex.13425

**Published:** 2022-01-10

**Authors:** Anthony W. Gilbert, Jeremy Jones, Maria Stokes, Carl R. May

**Affiliations:** ^1^ Therapies Department Royal National Orthopaedic Hospital Stanmore UK; ^2^ Faculty of Environmental and Life Sciences, School of Health Sciences University of Southampton Southampton UK; ^3^ NIHR Applied Research Collaboration North Thames UK; ^4^ NIHR Applied Research Collaboration Wessex UK; ^5^ Faculty of Public Health and Policy London School of Hygiene and Tropical Medicine London UK

**Keywords:** clinician preferences, COVID‐19, Normalisation Process Theory, organisation preferences, patient preferences, virtual consultations

## Abstract

**Aim:**

To investigate the experiences of patients, clinicians and managers during the accelerated implementation of virtual consultations (VCs) due to COVID‐19. To understand how patient preferences are constructed and organized.

**Methods:**

Semi‐structured interviews with patients, clinicians and managerial staff at a single specialist orthopaedic centre in the United Kingdom. The interview schedule and coding frame were based on Normalisation Process Theory. Interviews were conducted over the telephone or by video call. Abductive analysis of interview transcripts extended knowledge from previous research to identify, characterize and explain how patient preferences for VC were formed and arranged.

**Results:**

Fifty‐five participants were included (20 patients, 20 clinicians, 15 managers). Key mechanisms that contribute to the formation of patient preferences were identified. These were: (a) context for the consultation (normative expectations, relational expectations, congruence and potential); (b) the available alternatives and the implementation process (coherence, cognitive participation, collective action and reflexive monitoring). Patient preferences are mediated by the clinician and organisational preferences through the influence of the consultation context, available alternatives and the implementation process.

**Conclusions:**

This study reports the cumulative analysis of five empirical studies investigating patient preferences for VC before and during the COVID‐19 pandemic as VC transitioned from an experimental clinic to a compulsory form of service delivery. This study has identified mechanisms that explain how preferences for VC come about and how these relate to organisational and clinician preferences. Since clinical pathways are shaped by interactions between patient, clinicians and organisational preferences, future service design must strike a balance between patient preferences and the preferences of clinicians and organisations.

**Patient and Public Contribution:**

The CONNECT Project Patient and Public Involvement (PPI) group provided guidance on the conduct and design of the research. This took place with remote meetings between the lead researcher and the chair of the PPI group during March and April 2020. Patient information documentation and the interview schedule were developed with the PPI group to ensure that these were accessible.

## 1 INTRODUCTION

Virtual consultations (VCs), a collective term for phone and video consultations, received significant interest during the COVID‐19 pandemic. Their use allowed patients to access healthcare while avoiding close social contact. The COVID‐19 pandemic accelerated the implementation of the NHS Long Term Plan,^1^ which called for digitally enabled outpatient care across the NHS. The NHS *What Good Looks Like* framework^2^ provides guidance for health and care leaders to digitize services with a view to ‘improve the outcomes, experience and safety of our citizens.

In March 2020, the British government asked people to ‘stay at home’ and ‘protect the NHS’ as the COVID‐19 pandemic took hold. Many hospitals within the United Kingdom rapidly adopted VC to continue delivering healthcare while also adhering to social distancing guidelines. In May 2020, 185 NHS organisations were set up with the platform ‘Attend Anywhere’, and thousands of video consultations were carried out each day.^3^

VC is now central to the ongoing functions of patient care within the NHS in the United Kingdom. VCs have been shown to result in high levels of satisfaction^4^, ^5^ and to be a feasible method to maintain care during the pandemic.^6^, ^7^ The UK Government established guidance for face‐to‐face (F2F) assessments during COVID‐19,^8^ which included requirements for risk assessments, temperature checks, face coverings, hand sanitizer, social distancing, provision of personal protective equipment, cleaning after appointments and ventilation. The use of remote consultations before any in‐person contact was recommended during the pandemic.^9^ During ‘lockdown’, the opportunity for patients to have F2F care was limited.

Before the COVID‐19 pandemic, there was an accumulating evidence base around small, pilot‐stage projects of both telephone and video consultations across healthcare. A review of the literature, published in 2014, identified 27 published studies on the use of Skype (a software for video consultations) consultations with the majority of these being small pilot projects.^10^ Our previously published qualitative systematic review identified nine studies reporting the use of VC (both phone and video) in an orthopaedic rehabilitation setting before the pandemic. The majority of these were small projectsembedded within larger trials.^11^ The VOCAL study^12^ aimed to provide an in‐depth study of the advantages and limitations of video consultations across two contrasting clinical settings. Greenhalgh et al.^13^ provided a comprehensive overview of the complex challenges of embedding video consultations in practice. Much of the research published since the COVID‐19 pandemic investigates the acceptability of VC and the degree to which patients are satisfied with its use.^4^, ^5^

**Figure 1:**
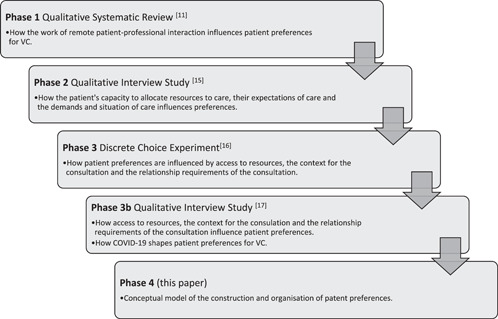
Overview of prior phases of the CONNECT Project research

This paper is the final phase of the CONNECT Project^14^; a mixed‐methods study that investigates patient preferences for VCs. The overall purpose of the project was to understand the potential interactions between patient preferences and the use of VC in orthopaedic rehabilitation (a summary of the different components of the project is given in Figure 1). Previous phases found that patient preferences for VC are influenced by the work patients themselves are required to do,^11^ their own situation and how this shapes their expectations about the use of VC.^15^ Patient preferences are influenced by whether they have access to the required resources to meet the requirements of the consultation.^16^ COVID‐19 appeared to influence preferences in favour of a VC but we cannot be sure whether this shift is permanent.^17^ This paper brings together these previous studies to develop a model of preference formation through an empirical investigation into the experiences of VC implementation due to COVID‐19.

To enable healthcare services to design pathways that enhance the uptake of the appropriate use of VC in clinical practice, it is important to understand how patients form their preferences. The aims of the study reported in this paper were to investigate the experiences of patients, clinicians and managers during the accelerated implementation of VC (both phone and video consultations) due to COVID‐19. The study aims to identify, characterize and explain how patient preferences to implement VC are decided and how they are organized following on from the COVID‐19 pandemic. The research question for this study was *‘how are patient preferences for VC decided and organised following COVID‐19?’* The protocol for the CONNECT Project was previously published.^14^

The study is informed by two theoretical perspectives.

1. Normalization Process Theory^18^ (NPT) provides an underpinning line of enquiry into the implementation process of VC.
2. Preference theory^19^ provides an understanding of how patient preferences are decided for VC.

Both NPT and Preference Theory rely on ideas about social and mental mechanisms to explain the outcomes of implementation processes and the production of preferences. Indeed, qualitative analysis of this problem must provide accounts of why phenomena occur^20^ and how these are motivated or shaped by different mechanisms. A mechanism can be defined as a process that ‘brings about or prevents change in a concrete system’,^21^ and that involves ‘constellation of activities and entities that are linked to one another in such a way that they regularly bring about a type of outcome’.^22^ These definitions underpin the work that follows.

## 2 METHODS

This paper is part of a larger body of work and forms Phase 4 of the CONNECT Project.

### 2.1 Setting

The research was conducted within a single specialist orthopaedic hospital in North London, UK. All participants were recruited from within the specialist hospital. The hospital had set a target of 80% VCs^7^ to reduce footfall and thus the risk of infection during the pandemic.

### 2.2 Participants

We aimed to recruit 20 patients, 20 clinicians and 15 managerial staff (including operational, improvement, administrative and clinical managers). We took a pragmatic approach to recruit an accessible sample of participants: For patients, we aimed to recruit at least 10 male patients and 10 female patients; for healthcare professionals, we aimed to recruit a range of occupational therapists and physiotherapists with experience of delivering VC; for managerial staff, we aimed to recruit a range of professionals with experience of being involved with the planning, set up and delivery of VC since the start of the pandemic. Participant inclusion and exclusion criteria are detailed in Table 1.

**Table 1:** Participant inclusion and exclusion criteria

Inclusion criteria	Exclusion criteria
Patients, over age 18 years, attending the research site for physiotherapy or occupational therapy Patients with experience of orthopaedic/musculoskeletal condition Patients able to provide informed written consent to enter the study Patients able to understand and speak English or a language covered by the RNOH Interpreter service Physiotherapists or occupational therapists (or assistants) who have delivered VC to treat patients with orthopaedic/musculoskeletal disorders Managerial staff (including clinical managers) with experience of VC	Patients without the capacity to consent Patients suffering from disorders other than orthopaedic as the primary cause (e.g., neurological or oncology disorders) Patients currently or previously treated by A. W. G. Staff members with no experience of VC

Abbreviation: VC, virtual consultation.

### 2.3 Recruitment

An emailed invitation to participate in the study was sent to all occupational therapists and physiotherapists with experience of using VC. Individuals within the organisation who had a role in the deployment of VC were invited to participate. Clinicians were asked to identify patients who were interested in participating. Once a patient had indicated they were happy to be approached, an email letter of invitation was sent to them, and they were asked to formally agree to be sent information about the study. Eligible and interested potential participants were provided with a participant information sheet and given at least 24 h to discuss the study with the researcher. They were enroled in the study upon informed consent, received by email, using a specifically designed email consent form.

### 2.4 Data collection

The interview schedule was developed based on NPT.^23^, ^24^, ^25^, ^26^ Definitions of the constructs of NPT can be seen in Tables 2 and 3. The full interview schedule can be seen in Appendix S1. Interviews were conducted using phone or video call. Interviews lasted around 60 min with the option to extend or shorten as required. All interviews were conducted by the same investigator (A. W. G., a male clinical research physiotherapist who is employed at the research site), and were audio‐recorded and transcribed verbatim.

**Table 2:** Integrative analysis of interview data PLEASE MOVE TABLE 2 TO THE RESULTS

Insights from the CONNECT Project research before COVID‐19 carried forward	New insights from this study after COVID‐19	Integrative analysis	
		New subconstruct	New construct
**Expectations** ^15^ − Experience of previous care − Perceived requirements of the session	**Normative expectations** ^26^ − Perceived safety and effectiveness of VC − Expectations about changes to the norms, rules and resources as a result of working with interventions and their components	Context (1) **Normative expectations**	**Context** for the consultation *(the circumstances that form the setting for the consultation)*
**Interactions** ^11^ − The expected and actual change in interactions due to VC	**Relational expectations** ^26^ − Perceived communication through VC use − Changes to the ways that people expect to be organized and relate to each other as a result of working with interventions and their components	Context (2) **Relational expectations**	
**Situation** ^15^ − The clinical status of the patient − The treatment and management required − The availability of healthcare to the patient **Expectations** ^15^ − The psychological status of the patient and the impact of VC delivery **Demands** ^15^ − Competing life demands **Context for the consultation** ^16^, ^17^ − Pathway related factors − Clinical and symptom‐related factors **Requirements of the consultation** ^16^, ^17^ − Objective factors − Interaction factors **Requirements** ^11^ − How the new processes required of VC (such as engaging from different places) fit in **COVID‐19** ^17^ − The impact of COVID‐19 on the delivery and availability of healthcare	**The usefulness of VC** − An understanding of the ability of VC to meet the needs of the appointment through experiential use − Ability to determine whether it was able to ‘fit in’ with their lifeworld **Plasticity** ^26^ − The extent to which interventions and their components are malleable and can be moulded to fit their contexts **Elasticity** ^26^ − The extent to which contexts can be stretched or compressed in ways that make space for interventions and their components and allow them to fit **External processes and events that shape patients access to resources to support VC** ^25^ − During COVID‐19, the option of in‐person care was removed and the only option was VC	Context (3) **Congruence**	
**Capacity** ^15^ − Financial resources − Access to material and informational resources − Support available through networks − Sources of healthcare capacity **Patients access to resources** ^16^, ^17^ − Socioeconomic factors − Access to, and willingness to engage with, VC **Resources** ^11^ − Ability to achieve the logistics of getting to a F2F or VC − Time available for care **Environment** ^11^ − Setting for physical rehabilitation − Setting for virtual rehabilitation − Access to hardware and software	**Internal processes and events that shape patients access to resources to support VC** ^25^ − Patient's access to hardware (such as phone or computer), up to date software to run the VC platform, adequate internet speed, the required rehabilitation equipment, the required space for rehabilitation and an understanding of how to get the most out of rehabilitation in the home **Internal processes and events that shape clinicians access to resources to support VC** ^25^ − Clinicians access to hardware and software and a confidential space to undertake a VC **Individual readiness** ^25^ − Patient and clinician readiness to translate individual beliefs and attitudes about VC into behaviours that are congruent, or not congruent, with (new) system norms and roles **Shared commitments** ^25^ − Patient and clinician readiness to translate shared beliefs and attitudes about VC into behaviours that are congruent, or not congruent, with (new) system norms and roles	Context (4) **Potential**	
**Expectations** ^15^ − Patient beliefs about the capability of VC	**Coherence** ^18^ − Coherence building that makes VC and its components meaningful: Participants contribute to enacting intervention components by working to make sense of its possibilities within their field of agency. They work to understand how intervention components are different from other practices, and they work to make them a coherent proposition for action	Implementation process (1) **Coherence**	**Implementation process** *(The translation of strategic intentions into routine practice)*
	**Cognitive participation** ^18^ − Cognitive participation that forms commitment around VC and its components: Participants contribute to enacting intervention components through work that establishes its legitimacy and that enrols themselves and others into an implementation process. This study frames how participants become members of a specific community of practice	Implementation process (2) **Cognitive participation**	
**Demands** ^15^ − The requirements of VC **Work** ^11^ − The required skills and expertize for a successful VC	**Collective action** ^18^ − Collective action through which effort is invested in VC and its components: Participants mobilize skills and resources and make VC workable. This study frames how participants realize and perform VC components in practice	Implementation process (3) **Collective action**	
**Demands** ^15^ − The things people need to do as a consequence of choice	**Reflexive monitoring** ^18^ − Reflexive monitoring through which the effects of VC and its components are appraised: Participants contribute to enacting intervention components through work that assembles and appraises information about their effects and utilize that knowledge to reconfigure social relations and action	Implementation process (4) **Reflexive monitoring**	

Abbreviations: F2F, for face‐to‐face; VC, virtual consultation.

**Table 3:** The mechanisms contributing to the formation of patient preference for VC

**Mechanism**				
**Normative expectations** *(The expected norms and rules of the consultation)*	**Relational expectations** *(The expected ways patients and clinicians relate to each other)*	**Congruence** *(How features of the consultation meet the requirements of the consultation)*	**Potential** *(Access to material and cognitive resources)*	**Implementation process**
*No, I think it's far more effective to have a face‐to‐face. They do as best they can, but there's limitations to having a 2D camera and being able to see in 3D, which obviously we see in 3D.* **[P4‐2]** *Can I do my rehabilitation virtually? Well, I don't know. You're the expert, you tell me. Yeah. I think if there was more information about—if it was rephrased and said how do I do my rehabilitation virtually? It's like well we do this, this and this. Then—yeah. Then I'm like okay, well they've thought about it, they know what they're doing and this is what we're going to do.* **[P4‐19]**	*I have been to a physio appointment before in another hospital and they did a whole assessment of my muscle strength and muscle balancing et cetera which is obviously not possible remotely.* **[P4‐1]** *There will be some where you absolutely, it's very straightforward, it's a straightforward pathway, you can easily do your first appointment virtually and that will be safe, through to those where you just could not do that because you've got to put your hands on, you've got to examine the person, you've got to watch them walk, et cetera.* **[N4‐1]**	*If I came in to see her, she wouldn't do any more. She would physically maybe touch me a little bit, but she wouldn't give me a massage or anything like I would ask her for. So it's not like there'd be any real gain for me physically by coming in. I would be a lot worse, just because I'd have to have driven and waited.* **[P4‐17]** *There are challenges with the initial assessment. With trauma patients I think you do pick up a lot about what's going on with them psychologically. Trying to pick that up on the screen is quite hard. So our pathway will probably stay the same for those initial face‐to‐face consultations.* **[N4‐10]**	*That I think is the nub of this is that I'm—in my position I'm quite happy with it but other people may feel they want to use equipment. Whether it's exercise machinery or a ball to sit on and get balancing or I don't know what else. What other equipment do you have in a physiotherapist department that you can't replicate at home? I mean that's the question I'm asking. * **[P4‐7]**	**Coherence** *(The work patients and clinicians do to make sense of the alternative consultation options)*
**Mechanism**				
**Normative expectations** *(The expected norms and rules of the consultation)*	**Relational expectations** *(The expected ways patients and clinicians relate to each other)*	**Congruence** *(How features of the consultation meet the requirements of the consultation)*	**Potential** *(Access to material and cognitive resources)*	**Implementation process**
*Based on my experience I think that I would like to have or to see maybe a first face‐to‐face in person assessment where maybe you can do other things that you cannot do virtually. Then, I think once decided is to have the choice of having the virtual follow‐ups possibly.* **[P4‐1]** *I swing from feeling like, no, this is a really unsafe way to work, we've got safeguarding, we've got suicide risks, all this kind of stuff, to then thinking, actually I'm sure there's some services that must be doing this and it's fine and we have systems to—you have SOPs set up, how to contact safeguarding, how to contact people who assess suicide.* **[C4‐13]**	*As I say, if I had only ever had virtual physio, so I'd seen the physio once in hospital and all the others were virtual, I wouldn't have felt as close.* **[P‐14]** *I think you get a lot more honesty in person…. When there's emotions and that involved, I think you get more—you get build a better relationship because that person is seeing you for real.* **[P4‐17]** *I think it's a lack of feeling that someone else is there that actually cares about you, by doing things virtually.* **[P4‐15]**	*Virtual's worked around my child care, because on Fridays I have my little one with me. If I had to start coming into hospital every Friday, I'd have to kind of source that childcare, make space for that hour journey to the hospital, while I'm waiting and then back again. For me, the difference between virtual and face‐to‐face is a big three hour difference of time. I can have my virtual appointment over the phone wherever I am, set up and go with it, and be done within half an hour, 45 minutes. * **[P4‐6]**	*The convenience is unreal. When you go to work four days a week, and you then you have to go to the hospital, come back, and I've got a kid as well, do you know, to fit everything around and travel back and forth, because the hospital for me is normally like an hour journey.* **[P4‐6]** *I think, from therapists, a fear of losing space. If we don't fill those face‐to‐face cubicles, what will happen? Will we lose that space? Will it then be taken away from us? Again, it's that balance, isn't it? That reassurance that you're not having—if we go one way, we're not going to be pushed that way.* **[C4‐6]**	**Cognitive participation** *(The work patients and clinicians do to invest commitment into the alternative consultation options)*
**Mechanism**				
**Normative expectations** *(The expected norms and rules of the consultation)*	**Relational expectations** *(The expected ways patients and clinicians relate to each other)*	**Congruence** *(How features of the consultation meet the requirements of the consultation)*	**Potential** *(Access to material and cognitive resources)*	**Implementation process**
*But it just kept cutting out, but I'm not sure whether that's her connection or whether it's my end connection. It was kind of annoying. But if it cut out she'd phone me or, as I say earlier, we started doing [x software], and it sort of worked better and did the trick.* **[P4‐16]** *But actually one thing that has come up to us from a team lead perspective has been about male therapists working with teenage girls, for example, and having to kind of get things [policies] in place.* **[N4‐12]**	*I remember I was moving from the sofa to by the window and sometimes I'd have to sit on the floor, just so the physio could see different bits of me. Then if they wanted to see me walking, then that's—well I don't know how well they could see me, but I imagine that it would be quite difficult.* **[P4‐18]** *But it just kept cutting out, but I'm not sure whether that's her connection or whether it's my end connection. It was kind of annoying. But if it cut out she'd phone me or, as I say earlier, we started doing [x software], and it sort of worked better and did the trick. So [x software] worked better.* **[P4‐16]**	*I live in [x location], so actually coming up to [x hospital] is a bit of a palaver and it's at least three hours on the train for me to come up. Six hours for an hour appointment or half an hour appointment is a bit of a trauma.* **[P4‐13]** *At the beginning because there was so much administration, and it completely depends on the therapist. One therapist will need more time to do admin stuff. I think we were trying to be quite gracious and give more time because it's also been, obviously because of the pandemic, a really stressful period and we're just—we haven't treated 12 patients or 15 patients a day. So, I think slowly we'll build that up. I think yeah, we've been more cautious.* **[C4‐16]**	*To get someone to try and get their phone round their back and show you where the pain is, that's the limitations that happen right here. You don't know if people have got access to someone else to hold to their phone for them while they turn around.* **[P4‐6]** *When things go very well is that they have somebody else to film for them. They have space. They've organised the area. They have dressed appropriately, and they have prepared well. They've got adequate space in their home environment.* **[C4‐7]**	**Collective action** *(The work patients and clinicians do to operationalise the alternative consultation options)*
**Mechanism**				
**Normative expectations** *(The expected norms and rules of the consultation)*	**Relational expectations** *(The expected ways patients and clinicians relate to each other)*	**Congruence** *(How features of the consultation meet the requirements of the consultation)*	**Potential** *(Access to material and cognitive resources)*	**Implementation process**
*At the beginning there was so much administration… One therapist will need more time to do admin stuff. I think we were trying to be quite gracious and give more time because it's also been, obviously because of the pandemic, a really stressful period and we're just—we haven't treated 12 patients or 15 patients a day. So, I think slowly we'll build that up. I think yeah, we've been more cautious.* **[C4‐16]** *I thought they were in this place and I thought they were doing this and exercise z and I saw them and they were worse than I thought they were. That has also frightened people—therapists I guess, thinking that, oh I thought they were better.* **[N4‐5]**	*I feel like I'm being held back just a little bit. I've gone from taking strides and going forward and I feel like I've gone back to baby steps a bit. That's not anybody's fault, it's just the situation.* **[P4‐4]** *A prime example is when I was telling [x physiotherapist] about, when I had done a lot of exercise or walking, I get an actual limp on my right leg, where my leg gives up a little bit. I really wanted to show her, but it was impossible to show her over the [x software], and that's why we left that as, next time I come in, I'll show you that.* **[P4‐6]**	*Like I say, I can't put a price on the time I've saved throughout all my hospital appointments. Six weeks of virtual has saved me probably 40 hours of travelling. When you work out how much time I would have spent—that's not even including petrol. So yeah, it's priceless.* **[P4‐6]** *What didn't work well was a chaotic environment at home, so other children involved would be just chaos.* **[C4‐4]** *One of those patients is coming back to see us as an outpatient, as a face‐to‐face. I don't think that it did meet her needs, actually, from a pain—I think she needed to be taken out of her environment which is quite challenging, quite chaotic and quite toxic at times.* **[C4‐12]**	*I mean like correcting someone's movements you can do it over the video, but I'm not sure how accurate that it. It could be accurate if the video quality is good, but less maybe if it's not that great. What else? I'm not sure.* **[P4‐1]** *You're reliant on someone's ability to be able to use the technology before you even get to know them.* **[P4‐6]** *So a couple of times I've had to mute [x software] and I've had to call their home phone. So I can hear their voice and see them on the screen.* **[C4‐1]**	**Reflexive monitoring** *(The work patients and clinicians do to appraise the alternative consultation options)*

Abbreviation: VC, virtual consultation.

### 2.5 Data management and analysis

Following transcription, the audio recordings were reviewed with the completed transcripts by AWG to enhance the familiarity with the content. The process was undertaken to review the content of the transcripts and to ensure all identifiable data were removed.

Interview transcripts were reviewed and uploaded into NVIVO (version 12). Data analysis followed the principles of abduction as set out by Tavory and Timmermans,^27^ described below:

1(a): Coding was initially undertaken in NVIVO by A. W. G. The concept of each line of the transcripts was identified and attributed to a description of the content. Attributions of content took the form of an NVIVO ‘node’. Nodes were arranged in relation to the coding manual, shown in Appendix S2. The final coding was reviewed by C. R. M.

1(b): Codes were characterized in light of the previously gained knowledge arising from the CONNECT Project in Phase I,^11^ Phase II^15^ and Phase III.^16^, ^17^ The purpose of the characterisation was to abductively extend insights from the previous research to develop new insights into the development and organisation of patient preferences.

2: Codes were then characterized in relation to the research question ‘*how are patient preferences for VC decided*’

3: Codes were subsequently characterized in relation to the research question ‘*how are patient preferences for VC organised*’

Reporting was conducted using the Standards for Reporting Qualitative Research^28^ (the report can be seen in Appendix S3).

### 2.6 Patient and public contribution

The CONNECT Project Patient and Public Involvement (PPI) group provided guidance on the conduct and design of the research. This took place with remote meetings between the lead researcher and the chair of the PPI group in March and April 2020, where it was decided an amendment should be submitted to NHS ethics to change the focus of the study to understand patients, clinicians and managers experiences of VC during COVID‐19. Patient information documentation and the interview schedule were developed with the PPI group to ensure that these were accessible.

## 3 RESULTS

Fifty‐five participants were included in the study: 20 patients (average age: 47 [range: 22–74], 10 female), 20 clinicians (14 physiotherapists, 17 female) and 15 managerial staff (11 female). Nine managerial staff consisted of managers situated within the Occupational Therapy and Physiotherapy Department and five managers who also had patient facing clinical care responsibilities. Six were managers situated across the entire hospital. The average interview length was 52 min (range: 19–70 min). All interviews were conducted over video call except for two patient interviews, which took place over the phone.

The study interviews took place between September and October 2020, between the UK ‘Lockdowns’ 1 & 2 due to COVID‐19. The patients within this study were forced to have VC due to the government restrictions and local Trust policy.

This study presents significant new data and performs an integrative analysis of this in relation to old data. The integrative analysis of previous and new insights is presented in Table 2. Interview extracts of participants' perspectives may be found in Table 3.

### 3.1 Coding and integrative analysis of interview data

Interview data were coded and characterized in relation to the previously identified factors that influence preference, identified from our earlier research. New insights were identified during this process. The integrative analysis led to the identification of factors that shape the formation of patient preferences for VC and are described below. The knowledge underpinning these factors from our previous research and new empirical data within this study are presented in Table 2.

#### 3.1.1 The context for the consultation

The context for the consultation is the circumstances that form the setting. This includes the expected standards and rules of care (normative expectations), the expected ways patients and clinicians are organized and relate to each other (relational expectations), the degree to which features of the consultation meet the requirements of the consultation (congruence) and the access to material and cognitive resources to support the consultation (potential).

##### 3.1.1.1 Normative expectations

Patients' expectations were founded on their previous experience of care. All patients within this study had experienced in‐person physiotherapy before and were able to speculate about the effectiveness of VC. The requirements of the consultation provided a reference point to understand the way VC would work for them. During COVID‐19, ‘stay at home’ became law and patients were satisfied with virtual care during this time and many were happy to not travel. The presence of COVID‐19 led to VC becoming the only way to access rehabilitation for the majority of patients and during this time patients in this study preferred VC to no care at all.

##### 3.1.1.2 Relational expectations

Patients had expectations about the ways patients and clinicians relate to each other during clinical interactions. Their previous experience of care provided a reference point to understand the changes in relationships with their clinicians over VC. Although many patients felt interactions over VC were inferior to F2F care, patients were willing to compromise and accept VC during COVID‐19.

##### 3.1.1.3 Congruence

The clinical status of the patient and the treatment required provided a point of departure to understand the ways the alternative consultation formats met their needs. Their needs could be shaped by a fluctuating clinical status, competing life demands, and the availability of healthcare. Each individual patient had varying degrees of ability to incorporate VC. Some patients found that VC was more easily incorporated into their life than an in‐person consultation and would consider using VC in the future beyond COVID‐19 because of this.

##### 3.1.1.4 Potential

Patients' access to resources shaped their ability to engage with virtual care. These resources included hardware (such as a phone, tablet or computer) and software (such as up‐to‐date operating software and the platform to undertake a video call). During the COVID‐19 pandemic, the platform *Attend Anywhere* was made available across the NHS in England. Resources were made available to patients to support the use of video calls.

#### 3.1.2 The implementation process of VC

Participants within this study were not offered the choice of a F2F consultation and all had to implement VC (either a telephone call or a video call with their clinicians). In these circumstances, a process of implementation took place. NPT provided the framework to build on previous iterations of the CONNECT Project to explain the implementation process for patients.^18^

##### 3.1.2.1 Coherence

Patients needed to understand the differences between VC and F2F. This was challenging during the pandemic when the introduction of VC was accelerated and individuals were inexperienced in VC as the main form of consultation. Clinical and administrative staff supported patients to understand the role of VC. The capabilities of VC were seen to be limited where an in‐person intervention was required, such as when hands on‐manual therapy or facilitated exercises were required. If a patient was concerned about their problem, they often felt that a thorough F2F assessment was preferable to a VC.

##### 3.1.2.2 Cognitive participation

In general, patients who found F2F attendance challenging were more committed to VC. For some, a traditional F2F appointment took significant planning and left the patient in pain due to their travel. Commitment for VC was enhanced with increased congruence for the patient. Many patients were concerned about catching COVID‐19 through travel to the hospital and this made the option of a VC preferable. Patients' willingness to use VC was shaped by their understanding of the benefits.

##### 3.1.2.3 Collective action

VC rehabilitation was challenging in the home environment for some patients. It was not possible to conduct the range of interventions that were often needed if the patient's video device was not portable. Mobile devices were helpful if, for instance, a patient had to film themselves walking upstairs or an occupational therapist needed to observe functional activities in the kitchen. Patients had to convey their symptoms over VC without the clinician being able to physically touch them.

The ‘work’ required of patients and clinicians over a VC was different from the ‘work’ of F2F care. Some patients and clinicians did not have the technical skills required to be able to use VC. Family members often supported patients with VC activities. Clinicians occasionally needed to teach patients the required computer skills over the phone. The burden of VC shaped preferences for ongoing use of VC.

##### 3.1.2.4 Reflexive monitoring

Patients were forthcoming with feedback about their experiences. Clinicians also discussed their own experiences to shape the virtual service. For instance, after several clinicians encountered technical challenges that interfered with the delivery of a VC, the virtual slots were increased from 30 min to one hour. Patients valued the extra time with their clinician and found this aspect of the VC to be beneficial. In response to these technical problems, clinicians made it clear to patients, at the start of a video call, that they would contact the patient via telephone if the VC cut out. As patients and clinician dyads experienced both VC and F2F, they were able to plan long‐term management, which often included the use of both VC and F2F.

Patients had set expectations about their own progress and were reluctant to engage a modality if they felt it was less effective than their preferred option. If a patient felt their progression was slower virtually, they preferred an F2F appointment. Some clinicians felt virtual assessments were less accurate than F2F; this viewpoint was further confirmed at follow‐up F2F appointments if a patient presented in a worse physical condition than was anticipated.

### 3.2 How preferences for VC are decided

Patient expectations provided the point of departure to make sense of the alternative consultation formats. These sense‐making activities shaped their willingness to implement the alternative consultation options. Patients had an awareness of what was required from the consultation and were able to determine whether a VC or a F2F would be a helpful format to achieve what was required. In this study, patients placed emphasis on the relational aspect of their care, whereas clinicians and managers placed more emphasis on the normative expectations of care. Patient expectations about the norms, rules and relationships with clinicians shaped their ability to implement the alternatives, which affected the way the alternative options were appraised.

Patients' ability to accommodate the consultation options shaped the way in which they made sense of their responsibilities and the value of the alternatives. Patients would determine whether a VC met their needs and this shaped their willingness to implement one format over another. During the pandemic, it was found that a traditional length consultation required additional administration for therapists, and this influenced clinicians' ability to do the required tasks to meet the objectives of the consultation. If a patient could successfully undertake a VC, this made available additional time and resources to spend doing usual day‐to‐day tasks because of the avoidance of travel. Patients and clinicians were able to determine the success of the consultation in relation to it meeting their needs and fitting in with their life. This shaped the way in which they appraised information about the alternative formats.

Patients' access to material and cognitive resources shaped the way in which they made sense of their responsibilities and the value of the alternatives, as well as their willingness to implement them. There was recognition that different individuals would have different access to resources. It was this level of access that shaped patients' ability to do the work of the alternatives. Some patients had access to adequate broadband and a device to be able to undertake VC and some had access to equipment and the space to be able to complete their rehabilitation in the home environment. Without these, successful implementation of VC was not possible and patients were more likely to prefer a F2F.

### 3.3 How preferences for VC are organized

Patient preferences were formed in the context of clinician and organisational preferences. The clinicians within this study were required to implement VC at a pace that required restructuring of policies and procedures. For many clinicians, the addition of VC worked well whereas for others VC was inferior to F2F.

The organisation invested heavily in resources for clinical staff to be able to undertake VC with patients. These additional resources shifted the context for clinicians in favour of undertaking VC. Patients arrived at the point of care with an established context of care; they had set expectations about what the norms and resources of care are and the relationship to their clinician. The congruence of the alternative care options and their access to cognitive and material resources were fixed and available alternatives for patients were restricted. When a clinician did not think that a VC would work, they would suggest a F2F, which influenced the patient's sense‐making of the alternatives and their commitment to VC. The work of implementing the alternatives for patients was shaped by the resources they could bring to bear; if they did not have access to adequate equipment, they were unable to do the required work to implement VC. In some circumstances, clinicians did not believe VC was appropriate.

Organisation and clinician context, the availability of alternatives and the work required of implementation directly influenced patient preferences and decision making.

## 4 DISCUSSION

This qualitative interview study is underpinned by NPT^18^ and Preference Theory.^19^ This study has extended the findings of our previous research through an investigation into patient, clinician and manager experience of the accelerated implementation of VC.

### 4.1 Strengths and limitations

A strength of this study is the cumulative abductive identification of insights through the different phases of the CONNECT Project, before,^11^, ^15^, ^16^ during^7^, ^29^ and after the COVID‐19 pandemic. The resulting model is the result of a pragmatic, real‐world investigation into the implementation of VC in practice. While we offer statements that may aid the prediction of preferences, further research is needed to understand their relative importance.

This study was conducted at a specialist London Hospital and focussed on orthopaedic rehabilitation and may not have applicability to other centres. To overcome this, a qualitative abductive analysis was conducted to identify more general factors that influence preference to allow for transportability within settings. The lead researcher (A. W. G.) is a healthcare professional within the centre, which could have limited the results through local familiarity. To mitigate this, patients who had a previous existing relationship with A. W. G. were excluded from the study. It was not possible, however, to exclude clinical staff, most of whom were known to A. W. G. This was considered in the data analysis through a process of defamiliarisation; attributions for each data point were orientated into a taxonomy to facilitate model development.

This investigation into patient preferences sought the experience of participants who did not always have a choice of consultation format due to COVID‐19. A limitation of this study is that the construction of preference in the context of COVID‐19 may not be representative of a post‐COVID world. A strength of the research is the variety of patients, clinicians and managerial staff included in the study provided a range of perspectives and context to support the development of the model. The use of Normalisation Process Theory provided focused attention towards key implementation factors that feed into the formation of preference.

### 4.2 Mechanistic model of preference formation

**Figure 2:**
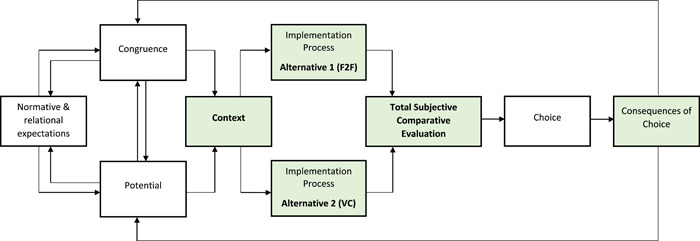
Model to explain the construction of patient preferences. F2F, face‐to‐face; VC, virtual consultation

Here, we present a theory of preference formation. A visual model to illustrate the formation of preferences has been developed from the integrative analysis and can be seen in Figure 2. We consider the formation of patient preferences as a mechanism. Our position is that patient preferences are the product of a total subjective comparative evaluation of the available options. The context for the consultation (normative expectations, relational expectations, congruence and potential), the available alternatives and the implementation process (coherence, cognitive participation, collective action, and reflexive monitoring) are all involved in shaping the total subjective comparative evaluation. These are the key entities that are linked to one another to form the construction of patient preferences.

1. Consultation Context
  Each individual patient context will present a unique potential to incorporate either a VC or a F2F for a clinical appointment. For some patients, the use of a VC will be burdensome; for others, the introduction of VC will be beneficial. Patients will need to have access to specific resources (the required hardware, software and skills to use these)^30^ to have a VC, particularly if VC is enforced. Patients will also need to be prepared to accept the change in their roles and responsibilities through VC use. A patient*'*s context is formed through the interactions between the level of resources they have at their disposal (their potential capacity), the degree to which the features of the consultation fit in with the circumstances (the congruence of the consultation alternatives), their expectations of what standard rehabilitation looks like (their normative expectations) and their expected interactions with their clinician (their relational expectations) of enroling in a F2F or VC. If the patient context lends itself to one consultation being more beneficial than the other, they will prefer the most beneficial consultation.
2. The implementation process of VC and F2F
  The patient context dictates the work required of patients to implement the available alternatives. Patients need to make sense of the differences between the consultation formats and build an understanding of the potential alternatives. Clinician and Organisational sense‐making shapes patient sense‐making. Patients must invest commitment and engage in the process of determining which alternative is more beneficial and define what they need to do. Each alternative will require different tasks and patients may need to acquire new skills. Patients need to collect and appraise information about the effects of each consultation alternative. These mechanisms are underpinned by the patient context. The implementation process shapes the total subjective comparative evaluation of the alternatives.
3. The formation of preference
  A total subjective comparative evaluation is undertaken by the patient. The patient will consider all the available information and choose the alternative, which brings them the most benefit. The patient will prefer the option that yields the most benefit.
4. The consequences of choice

The choice a patient makes will have a range of consequences on their context, their implementation process, and their overall preferences. The outcomes and consequences will differ for each individual patient, as this is all dependent on their individual context.

A patient is more likely to implement a preferable alternative of care. This understanding of the mechanisms that influence preference formation is helpful to understand implementation processes.

### 4.3 Results in context

This present research study builds on the previous insights gained from earlier phases of the CONNECT Project^11^, ^15^, ^16^, ^17^ to understand how patient preferences for VC have changed during the accelerated implementation of VC during the COVID‐19 pandemic.

The COVID‐19 pandemic led to the restructuring of normative and relational expectations of care. The emergency nature of care led to a shift in the perceptions of how healthcare should be judged. While many patients acknowledged, within this study, that ordinarily they would have preferred in‐person care, they were satisfied and grateful for the opportunity of VC as the only option for rehabilitation. Even if in‐person care was available, the introduction of the national lockdown restricted in‐person care and some were unable to travel because of medical reasons. For these patients, VC via telephone or video was the only option and VC was preferable to the alternative (no care).

Healthcare organisations in England were provided with a platform to deliver VC. Organisations invested effort to help patients to understand the role of VC.^31^ Resources that aid sense‐making, such as our previously developed sensitizing questions to aid preference formation,^15^ are likely to help patients understand the value of VC.

Historically, standard care demanded in‐person rehabilitation appointments. For most patients in this study, the standard of care became VC during the pandemic.^7^ Patients and clinicians developed skills and expertise with virtual interactions. Patient and clinician sense‐making and commitment to VC improved during the pandemic and clinicians provided ongoing support for patients to make the demands of care easier. Patients valued the support of clinicians and administrative staff to understand how VC worked and for their assistance with technical problems.^29^

The implementation of VC may pose several challenges for patients. For example, people with disabilities are less likely to have a suitable infrastructure.^32^ Within this present study, this infrastructure included access to hardware and up‐to‐date software and the space to undertake rehabilitation in the home environment. If a patient cannot undertake a VC because of a lack of infrastructure, they are more likely to value an in‐person consultation.

The use of VC may be more challenging for patients with communication barriers, patients with a lack of education, those with language or literacy barriers or those with intellectual disabilities.^32^ Patient satisfaction is positively associated with technical performance,^33^ and in our study, clinicians often had to support patients with technical challenges. Some patients did not possess the technical skills to use VC,^7^ which reflects the nationwide picture.^30^ While this clinician support may have a positive impact on patient experience, this will reduce the overall resources of the clinical team to be able to provide rehabilitation for patients.

Communicating over VC placed greater emphasis on verbal communication skills during these interactions.^34^ Failed VC was deemed to occur when there were issues with communication.^6^ In addition, clinicians needed to be able to trust the VC—many orthopaedic professionals lost confidence with virtual calls when issues arose.^35^ Clinicians' normative expectations of undertaking a thorough hands‐on assessment were important, many feared missing sinister pathology and screening of ‘red flags’^36^ or ‘safety netting’^37^ may be a useful way to overcome these concerns. People need to commit to using VC, sharing of good news stories^29^, ^38^ might help influence the views of clinicians who are reluctant to engage with telehealth.^39^

Shared decision‐making, where clinicians and patients make decisions in partnership using the best available evidence, must be considered in light of different power relationships. The Agency model of power^40^ suggests ‘ontologically autarchic’ individuals hold power. In the context of a patient and clinician relationship, the clinician (situated within the organisation) is perceived as having power while patients perceive themselves as relatively powerless.^41^ Power is exercised *‘by making others do things that they would not otherwise do, or by resisting the attempts of others to make them do such things as would be against their preferences*’.^42^

**Figure 3:**
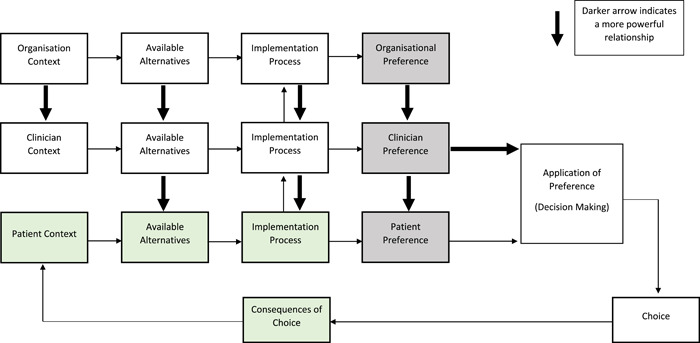
Map of empirical data of patient preferences in the context of organisational and clinician preferences

Organisations and clinicians have a role in helping patients to understand the role of VC and some of the ways in which organisations and clinicians can influence patient preferences are shown in Table 4. The application of preferences and decision‐making may take place as a shared decision, where patients and clinicians have equal power, or the more powerful individuals may exert their own preferences to enable preferable outcomes (Figure 3). Consideration of these mechanisms will facilitate shared decision‐making in practice.

**Table 4:** The impact of organisation and clinician preferences on patient preferences

Mechanism	Impact of organisation and clinician preferences
Normative expectations	Establish the norms and rules for care
Relational expectations	Establish the ways in which patients and clinicians are organized and relate to each other
Congruence	Can restrict or develop care pathways that are more easily accommodated in the patient's lifeworld
Potential	Can withhold or provide access to material and informational resources to patients
Coherence	Can frame the ways patients make sense of the alternative consultation options
Cognitive participation	Can withhold or support patients to invest commitment into the alternative consultation options
Collective action	Can make it harder or easier for patients to operationalize the alternative consultation options
Reflexive monitoring	Can frame the ways patients appraise the alternative consultation options

The NHS Long Term Plan^1^ set out a vision for a digital NHS but the COVID‐19 pandemic led to a ‘big bang’ of technological change^43^ where services rapidly converted F2F to VC in line with government guidelines. The timescale for the relaxation of social distancing restrictions in the UK remains uncertain; the capacity for F2F clinics will continue to be reduced during this period. Predicted modelling suggests that up to 28 million operations were cancelled or postponed globally during the first wave of COVID‐19^44^ and orthopaedics is now facing a substantial backlog of surgical cases.^45^ There is likely to be an ongoing reliance and pressure to use VC as remote consultations have been proposed as a potential way to increase capacity in orthopaedics.^46^ This pressure will continue to influence clinician and patient preferences. Healthcare must, therefore, be sensitive to clinician and organisational preferences. Clinicians need to develop sensitive ways to manage the ‘arenas of struggle’^47^ between high‐ and low‐powered individuals when preferences are incongruent. Agreements between healthcare professional and patient preferences are more likely to lead to successful uptake and adherence to modalities that patients conclude to be more beneficial.

Within our theoretical model, a patient will prefer the alternative that brings them the most benefit. Patient preferences are shaped by the context of the consultation and the implementation process of the alternatives. While this theoretical model was underpinned by empirical data of virtual orthopaedic rehabilitation consultations, this model is transportable to other areas of healthcare. It can be applied across a range of domains of healthcare delivery format, which may include preferences for virtual appointments across other sectors of healthcare, or preferences for different treatment modalities. Such a model could also be used to explain the empirical challenges of adherence to treatment regiments and management programmes when patients are offered a choice.

## 5 CONCLUSIONS

This was an empirical investigation into the experiences of patients, clinicians and healthcare managers during the accelerated implementation of VC during COVID‐19. This study has explained patient preferences through the accumulation of several pieces of work as VC changed from an experimental clinic to a compulsory form of service delivery during the COVID‐19 pandemic. This study presents a robust conceptual model of preference formation.

Patient preferences are decided in the form of a total subjective comparative evaluation of the available alternatives of care. This study found that the implementation process of investing meaning, commitment, effort and comprehension into the available options informed the total subjective comparative evaluation and the formation of preference. The preferences of clinicians and the organisation need to be considered as these were shown to mediate patient preferences. Since decision‐making will take place in the context of patient's, clinician's and organisations' preferences, future pathway design should be sensitive to patient preferences while acknowledging the preferred outcomes of clinicians and organisations.

## CONFLICT OF INTERESTS

The authors declare that there are no conflict of interests.

## ETHICS STATEMENT

Ethical approval was received for this study (Approval received 4 December 2018 from the South Central‐Oxford C Research Ethics Committee [IRAS ID: 255172, REC Reference 18/SC/0663]). An amendment was made to investigate the experiences of VC on 7 April 2020. All participants were provided with a participant information sheet and given at least 24 h to consider the information and ask questions before being recruited into the study. All participants provided informed, written consent before enrolment.

## AUTHOR CONTRIBUTIONS

Anthony W. Gilbert wrote the paper and conceived the project with Carl R. May, Jeremy Jones and Maria Stokes. Carl R. May guided qualitative data collection. Anthony W. Gilbert conducted all the interviews. Carl R. May assisted with data analysis, and with Anthony W. Gilbert developed the model. Carl R. May, Jeremy Jones and Maria Stokes edited and critically revised the paper. All authors have read and approved the manuscript.

## Supporting information

Supporting information.

Supporting information.

Supporting information.

## Data Availability

Materials from the research are available under reasonable request to the corresponding author.

## References

[1] NHS . 2019. The NHS Long Term Plan; 2019. Accessed November 15, 2021. Available from: https://www.longtermplan.nhs.uk/wp-content/uploads/2019/08/nhs-long-term-plan-version-1.2.pdf

[2] NHSx. 2021. What Good Looks Like framework. Accessed September 30, 2021. https://www.nhsx.nhs.uk/digitise-connect-transform/what-good-looks-like/what-good-looks-like-publication/

[3] Rapson J . COVID sparks boom in digital hospital outpatient appointments. Health Services Journal. 2021. https://www.hsj.co.uk/technology-and-innovation/covid-sparks-boom-in-digital-hospital-outpatient-appointments/7027590.article. Accessed November 15, 2021.

[4] Vusirikala A , Ensor D , Asokan AK , et al. Hello, can you hear me? Orthopaedic clinic telephone consultations in the COVID‐19 era‐ a patient and clinician perspective. World J Orthop. 2021;12(1):24‐34. 10.5312/wjo.v12.i1.24 33520679 PMC7814312

[5] Rizzi AM , Polachek WS , Dulas M , Strelzow JA , Hynes KK . The new ‘normal’: Rapid adoption of telemedicine in orthopaedics during the COVID‐19 pandemic. Article. Injury. 2020;51(12):2816‐2821. 10.1016/j.injury.2020.09.009 32951916 PMC7493795

[6] Kumar S , Kumar A , Kumar M , Kumar A , Arora R , Sehrawat R . Feasibility of telemedicine in maintaining follow‐up of orthopaedic patients and their satisfaction: a preliminary study. J Clin Orthop Trauma. 2020;11(suppl 5):S704‐S710. 10.1016/j.jcot.2020.07.026 32837105 PMC7395587

[7] Gilbert AW , Billany JCT , Adam R , et al. Rapid implementation of virtual clinics due to COVID‐19: report and early evaluation of a quality improvement initiative. BMJ Open Qual. 2020;9(2):9. 10.1136/bmjoq-2020-000985 PMC7247397 32439740

[8] Pensions DfWa . Guidance for health and disability assessment providers carrying out face‐to‐face assessments during the COVID‐19 period. Gov.UK. Accessed June 20, 2021. https://www.gov.uk/government/publications/carrying-out-a-face-to-face-health-assessment-during-covid-19-guidance-for-assessment-providers/guidance-for-health-and-disability-assessment-providers-carrying-out-face-to-face-assessments-during-the-covid-19-period

[9] Physiotherapy CSo . COVID‐19: guide for rapid implementation of remote physiotherapy delivery. Accessed June 20, 2021. https://www.csp.org.uk/publications/covid-19-guide-rapid-implementation-remote-physiotherapy-delivery

[10] Armfield NR , Bradford M , Bradford NK . The clinical use of Skype—for which patients, with which problems and in which settings? A snapshot review of the literature. Int J Med Inform. 2015;84(10):737‐742. 10.1016/j.ijmedinf.2015.06.006 26183642

[11] Gilbert A , Jones J , Jaggi A , May C . Use of virtual consultations in an orthopaedic rehabilitation setting: how do changes in the work of being a patient influence patient preferences? A systematic review and qualitative synthesis. BMJ Open. 2020;10:e036197. 10.1136/bmjopen-2019-036197 PMC7497523 32938591

[12] Greenhalgh T , Vijayaraghavan S , Wherton J , et al. Virtual online consultations: advantages and limitations (VOCAL) study. BMJ Open. 2016;6. 10.1136/bmjopen-2015-009388 PMC4735312 26826147

[13] Greenhalgh T , Shaw S , Wherton J , et al. Real‐world implementation of video outpatient consultations at macro, meso, and micro levels: mixed‐method study. Article. J Med Internet Res. 2018;20(4):150. 10.2196/jmir.9897 PMC5930173 29625956

[14] Gilbert A , Jones J , Stokes M , Mentzakis E , May CR . Protocol for the CONNECT Project: a mixed methods study investigating patient preferences for communication technology use in orthopaedic rehabilitation consultations. BMJ Open. 2019;9(12):e035210. 10.1136/bmjopen-2019-035210 PMC6924859 31831552

[15] Gilbert AW , Jones J , Stokes M , May CR . Factors that influence patient preferences for virtual consultations in an orthopaedic rehabilitation setting: a qualitative study. BMJ Open. 2021;11(2):e041038. 10.1136/bmjopen-2020-041038 PMC7908916 33632750

[16] Gilbert AW , Mentzakis E , May CR , Stokes M , Jones J . Patient preferences for use of virtual consultations in an orthopaedic rehabilitation setting: Results from a discrete choice experiment. J Health Serv Res Policy. 2021. 10.1177/13558196211035427 PMC8772015 34337980

[17] Gilbert A , May C , Brown H , Stokes M , Jones J . A qualitative investigation into the results of a discrete choice experiment and the impact of COVID‐19 on patient preferences for virtual consultations. Arch Physiother. 2021. Under Review.11(1):1–13. 34488898 10.1186/s40945-021-00115-0 PMC8419808

[18] May C , Rapley T , Finch T . Normalization Process Theory. In: Nilsen P , Birken S , eds. International Handbook of Implementation Science. Edward Elgar; 2020:144‐167.

[19] Hausman DM . Preference, Value, Choice, and Welfare. Cambridge University Press; 2012.

[20] Hechter M , Horne H . Theories of social order: a reader. Stanford Social Sciences. 2nd ed. Stanford University Press; 2009.

[21] Bunge M . How does it work? The search for explanatory mechanisms. Philos Soc Sci. 2004;34(2):182‐210. 10.1177/0048393103262550

[22] Hedstrom P . Dissecting the Social: On the Principles of Analytical Sociology. Cambridge University Press; 2005.

[23] May C , Finch T . Implementing, embedding, and integrating practices: an outline of Normalization Process Theory. Sociology. 2009;43(3):535. 10.1177/0038038509103208

[24] May C . A rational model for assessing and evaluating complex interventions in health care. BMC Health Serv Res. 2006;6. 1–11. 10.1186/1472-6963-6-86 16827928 PMC1534030

[25] May C . Towards a general theory of implementation. Implement Sci. 2013;8. 1–14. 10.1186/1748-5908-8-18 23406398 PMC3602092

[26] May CR , Johnson M , Finch T . Implementation, context and complexity. Implement Sci. 2016;11. 1–12. 10.1186/s13012-016-0506-3 27756414 PMC5069794

[27] Tavory I , Timmermans S . Abductive Analysis: Theorizing Qualitative Research. University of Chicago Press; 2014:172‐172.

[28] O'Brien BC , Harris IB , Beckman TJ , Reed DA , Cook DA . Standards for reporting qualitative research: a synthesis of recommendations. Acad Med. 2014;89(9):1245‐1251. 10.1097/ACM.0000000000000388 24979285

[29] Ernst A , Schlattmann P , Waldfahrer F , Westhofen M . Leadership reflections a year on from the rapid roll‐out of virtual clinics due to COVID‐19: a commentary. BMJ Leader. 2021;5:1‐5. 10.1136/leader-2020-000363

[30] Castle‐Clarke S . What will new technology mean for the NHS and its patients? Four big technological trends. https://www.nuffieldtrust.org.uk/files/2018-06/1530028974_the-nhs-at-70-what-will-new-technology-mean-for-the-nhs-and-its-patients.pdf

[31] Letocha J 2020. RNOH virtual outpatient clinics: a patient guide (Royal National Orthopaedic Hospital version). Accessed November 15, 2020. https://youtu.be/1aL-WFe82xo

[32] Annaswamy TM , Verduzco‐Gutierrez M , Frieden L . Telemedicine barriers and challenges for persons with disabilities: COVID‐19 and beyond. Disabil Health J. 2020;13(4):100973. 10.1016/j.dhjo.2020.100973 32703737 PMC7346769

[33] Bradwell H , Baines R , Edwards K , et al. Exploring patient and staff experiences of video consultations during COVID‐19 in an English outpatient care setting: secondary data analysis of routinely collected feedback data. medRxiv. 2021. 10.1101/2020.12.15.20248235 PMC8989384 35311688

[34] Roberts LC , Osborn‐Jenkins L . Delivering remote consultations: talking the talk. Musculoskelet Sci Pract. 2020;52:102275. 1–7. 10.1016/j.msksp.2020.102275 33132068 PMC7573651

[35] Gilbert AW , Booth G , Betts T , Goldberg A . A mixed‐methods survey to explore issues with virtual consultations for musculoskeletal care during the COVID‐19 pandemic. BMC Musculoskelet Disord. 2021;22(1):245. 10.1186/s12891-021-04113-y 33673844 PMC7933396

[36] Paling C . The complex problem of identifying serious pathology in musculoskeletal care: managing clinical risk during the COVID pandemic and beyond. Musculoskelet Sci Pract. 2021;54:102379. 10.1016/j.msksp.2021.102379 34052175

[37] Greenhalgh S , Finucane LM , Mercer C , Selfe J . Safety netting; best practice in the face of uncertainty. Musculoskelet Sci Pract. 2020;48:102179. 10.1016/j.msksp.2020.102179 32560875 PMC7214294

[38] Wherton J , Shaw S , Papoutsi C , Seuren L , Greenhalgh T . Guidance on the introduction and use of video consultations during COVID‐19: important lessons from qualitative research. BMJ Leader. 2017;96:519‐521. 10.1136/leader-2020-000262

[39] Malliaras P , Merolli M , Williams CM , Caneiro JP , Haines T , Barton C . ‘It's not hands‐on therapy, so it's very limited’: Telehealth use and views among allied health clinicians during the coronavirus pandemic. Musculoskelet Sci Pract. 2021;52. 10.1016/j.msksp.2021.102340 PMC7862900 33571900

[40] Terence B . Two concepts of coercion. Theory Soc. 1978;5(1):97‐112. 10.1007/BF01880862

[41] Lask B . Patient–clinician conflict: causes and compromises. J Cyst Fibros. 2003;2(1):42‐45. 10.1016/S1569-1993(03)00006-7 15463846

[42] Clegg S . Frameworks of Power. Sage; 2002.

[43] Carroll N , Conboy K . Normalising the “new normal”: Changing tech‐driven work practices under pandemic time pressure. Int J Inf Manage. 2020;55:102186. 10.1016/j.ijinfomgt.2020.102186 32836643 PMC7358767

[44] COVIDSurg Collaborative . Elective surgery cancellations due to the COVID‐19 pandemic: global predictive modelling to inform surgical recovery plans. Br J Surg. 2020;107(11):1440‐1449. 10.1002/bjs.11746 32395848 PMC7272903

[45] Jain A , Jain P , Aggarwal S . SARS‐CoV‐2 impact on elective orthopaedic surgery: implications for post‐pandemic recovery. J Bone Joint Surg Am. 2020;102(13):e68. 10.2106/JBJS.20.00602 32618916 PMC7396217

[46] Carr A , Smith JA , Camaradou J , Prieto‐Alhambra D . Growing backlog of planned surgery due to COVID‐19. BMJ. 2021;372:n339. 10.1136/bmj.n339 33563590

[47] Hindess B . Power, interests and the outcomes of struggles. Sociology. 1982;16(4):498‐511.

