# Supplementary material for: Patient, clinician and manager experience of the accelerated implementation of virtual consultations following COVID‐19: A qualitative study of preferences in a tertiary orthopaedic rehabilitation setting
Source: Health Expect. 2022 Jan 10;25(2):775–90. doi: 10.1111/hex.13425 (PMC8957728; doi:10.1111/hex.13425)
Supplement: Supplementary file 1 — Supporting information. [file HEX-25--s001.docx]

**NPT Model**

**Interview Guide**

*General Intro*

- What were your experiences of VC

*Implementation*

- Coherence
  - How is VC different from F2F
  - What are the benefits of VC
  - How is it different preparing for VC than F2F
- Cognitive Participation
  - Tell me about how you use VC
  - Do you think VC works for you
- Collective Action
  - Wat do you need to do with a VC to get it set up / use
  - Are you confident with VC – probe
  - What skills do you need to have
  - How were you supported / what support do you need
- Reflexive monitoring
  - Is VC effective
  - How do we need to think about its usefulness, what works for you
  - How was your experience of VC
  - What do we need to change

*Embedding*

- Potential
  - How does your clinician see VC
  - What things shape whether you can do it or not? What do you need?
- Outcomes
  - How does it change what you need
  - How does it shape relationships
  - What makes it success / fail?
- Context
  - How do you make it fit in with your life?

*Integration*

- - How do you see VC working at this hospital / elsewhere

*Preference*

- - Did VC work for you
  - What do you like about it
  - What do you like about F2F
  - If you were asked to use it again, what would you choose? Why?
