# Supplementary material for: Patient, clinician and manager experience of the accelerated implementation of virtual consultations following COVID‐19: A qualitative study of preferences in a tertiary orthopaedic rehabilitation setting
Source: Health Expect. 2022 Jan 10;25(2):775–90. doi: 10.1111/hex.13425 (PMC8957728; doi:10.1111/hex.13425)
Supplement: Supplementary file 2 — Supporting information. [file HEX-25--s002.docx]

| **NPT construct and source references** | | **Standard**  **acronym** |
| --- | --- | --- |
| **Coherence** | Coherence building that makes interventions and their components meaningful: participants contribute to enacting intervention components by working to make sense of its possibilities within their field of agency. They work to understand how intervention components are different from other practices, and they work to make them a coherent proposition for action ^1^. | CO |
|  | **Differentiation**: An important element of sense-making work is to understand how interventions and their components, and prior practice are different from each other. ^2^ | CODI |
|  | **Communal specification**: Sense-making relies on people working together to build a shared understanding of the aims, objectives, and expected benefits of interventions and their components. ^2^ | COCS |
|  | **Individual specification**: Sense-making has an individual component, too. Here participants in coherence work need to do things that will help them understand their specific tasks and responsibilities around interventions and their components. ^2^ | COIS |
|  | **Internalization**: Sense-making involves people in work that is about understanding the value, benefits and importance of interventions and their components. ^2^ | COIN |
| **Cognitive Participation** | Cognitive participation that forms commitment around an intervention and its components: participants contribute to enacting intervention components through work that establishes its legitimacy and that enrols themselves and others into an implementation process. This work frames how participants become members of a specific community of practice ^1^ | CP |
|  | **Initiation**: A core problem is whether or not key participants are working to drive interventions and their components forward. ^2^ | CPIN |
|  | **Enrolment**: Participants may need to organize or reorganize themselves and others in order to collectively contribute to the work involved in interventions and their components. ^2^ | CPEN |
|  | **Legitimation**: An important component of relational work around interventions and their components is the work of ensuring that other participants believe it is right for them to be involved, and that they can make a valid contribution to it*.* ^2^ | CPLE |
|  | **Activation**: Once it is underway, participants need to collectively define and enact the actions and procedures needed to sustain interventions and their components and to stay involved. ^2^ | CPAC |
| **Collective Action** | Collective action through which effort is invested in an intervention and its components: participants mobilize skills and resources and make a complex intervention workable. This work frames how participants realize and perform intervention components in practice ^1^. | CA |
|  | **Interactional Workability**: This refers to the interactional work that people do with each other, and with the components of interventions and their components when they seek to operationalize them in everyday settings. ^3^ | CAIW |
|  | **Relational Integration**: This refers to the knowledge work that people do to build accountability and maintain confidence in interventions and their components and in each other as they use them. ^3^ | CARI |
|  | **Skill set Workability**: This refers to the allocation and training work that underpins that is built up around interventions and their components as it is operationalised in the real world. ^3^ | CASW |
|  | **Contextual Integration**: This refers to the resource work – supporting interventions and their components through the allocation of different kinds of resources and the execution of protocols, policies and procedures. ^3^ | CACI |
| **Reflexive Monitoring** | Reflexive monitoring through which the effects of an intervention and its components are appraised: participants contribute to enacting intervention components through work that assembles and appraises information about their effects and utilize that knowledge to reconfigure social relations and action ^1^. | RM |
|  | **Systematization**: participants in interventions and their components may seek to determine how effective and useful it is for them and for others, and this involves the work of collecting structured information in a variety of ways. ^2^ | RMSY |
|  | **Communal appraisal**: participants work together - sometimes in formal collaboratives, sometimes in informal groups to evaluate the worth of interventions and their components They may use many different means to do this drawing on a variety of experiential and systematized information. ^2^ | RMCA |
|  | **Individual appraisal**:  Participants in interventions and their components also work experientially as individuals to appraise its effects on them and the contexts in which they are set. From this work stem actions through which individuals express their personal relationships to new technologies or complex interventions. ^2^ | RMIA |
|  | **Reconfiguration**: appraisal work by individuals or groups may lead to attempts to redefine procedures or modify interventions and their components^2^ | RMRE |
| **Contextual features of interventions and their components** | Contexts provide social structural and social cognitive resources, and we can frame these in relation to different mechanisms. [These] are concerned with any context’s capacity to accommodate implementation processes ^1^. | CF |
|  | **Plasticity**: The extent to which interventions and their components are malleable and can be moulded to fit their contexts. ^4^ ^5^ | CFPL |
|  | **Elasticity**: The extent to which contexts can be stretched or compressed in ways that make space for interventions and their components and allow them to fit. ^5^ | CFEL |
|  | **Coupling**: Relations of interdependence between people, interventions and their components, and the contexts in which they are working. ^5 6^ | CFCO |
| **Negotiated outcomes** | NPT characterizes contexts as dynamic; an important prediction of the theory is that how participants in implementation processes relate to other actors, processes and structures to be found in their environments really does matter ^1^. | NO |
|  | **Norms and Normative restructuring:** Changes to professional norms, rules and resources as a result of working with interventions and their components ^5^ | NONO |
|  | **Roles and Relational restructuring:** Changes to the ways that people are organised and relate to each other as a result of working with interventions and their components ^5^ | NORO |
|  | **Integration:** Variable reproduction and sustainment of enacted intervention components over time and between settings ^2^ | NOIN |
|  | **Intervention success:** experienced workability and integration in contexts ^4^ | NOIS |
| **Potential** | [These are] concerned with the ways in which participants relate to the activities involved in implementing intervention components. These are social cognitive resources ^1^. | PO |
|  | **Individual readiness:** Participants’ readiness to translate individual beliefs and attitudes about interventions and their components into behaviours that are congruent, or not congruent, with (new) system norms and roles. ^7^ | POIR |
|  | **Shared commitments:** Participants’ readiness to translate shared beliefs and attitudes about interventions and their components into behaviours that are congruent, or not congruent, with (new) system norms and roles. ^7^ | PISC |
|  | **External (exogenous) processes and events** that shape the capacity of participants to implement and operationalize interventions and their components (e.g. Austerity, Covid) ^7^ | PIEX |
|  | **Internal (endogenous) processes and events** that shape the capacity of participants to implement and operationalize interventions and their components (e.g. staff shortages, strategic initiatives) ^7^ | PIEN |

1. May C, Rapley T, Finch T. Normalization Process Theory. In: Nilsen P, Birken S, eds. International Handbook of Implementation Science. London: Edward Elgar 2020:144-67.

2. May C, Finch T. Implementation, embedding, and integration: an outline of Normalization Process Theory. *Sociology* 2009;43(3):535-54.

3. May C. A rational model for assessing and evaluating complex interventions in health care. *Bmc Health Serv Res* 2006;6(86 ):1-11. doi: doi:10.1186/1472-6963-6-86

4. May C. Agency and implementation: Understanding the embedding of healthcare innovations in practice. *Social Science & Medicine* 2013;78(0):26-33. doi: <http://dx.doi.org/10.1016/j.socscimed.2012.11.021>

5. May CR, Johnson M, Finch T. Implementation, context and complexity. *Implement Sci* 2016;11(1):141. doi: 10.1186/s13012-016-0506-3

6. Perrow C. Normal accidents : living with high-risk technologies. New York: Basic Books 1984.

7. May C. Towards a general theory of implementation. *Implement Sci* 2013;8(1):18.
