# Supplementary material for: Patient, clinician and manager experience of the accelerated implementation of virtual consultations following COVID‐19: A qualitative study of preferences in a tertiary orthopaedic rehabilitation setting
Source: Health Expect. 2022 Jan 10;25(2):775–90. doi: 10.1111/hex.13425 (PMC8957728; doi:10.1111/hex.13425)
Supplement: Supplementary file 3 — Supporting information. [file HEX-25--s003.docx]

# Appendix 3 – SRQR Checklist

# Patient preferences for virtual consultations in the transition from COVID-19: A qualitative study

|  | **Item** | **Page** |
| --- | --- | --- |
| Title | Item 1. Title: Concise description of the nature and topic of the study. Identifying the study as qualitative or indicating the approach (e.g., ethnography, grounded theory) or data collection methods (e.g., interview, focus group) is recommended. | 1 |
| Abstract | Item 2. Abstract: Summary of key elements of the study using the abstract format of the intended publication; typically includes background, purpose, methods, results, and conclusions. | 1 |
| Problem Formulation | Item 3. Problem Formulation: Description and significance of the problem/phenomenon studied; review of relevant theory and empirical work; problem statement. | 2 |
| Purpose or research question | Item 4. Purpose or research question: Purpose of the study and specific objectives or questions. | 2 |
|  | Item 5. Qualitative approach and research paradigm: Qualitative approach (e.g., ethnography, grounded theory, case study, phenomenology, narrative research) and guiding theory if appropriate; identifying the research paradigm (e.g., post-positivist, constructivist/interpretivist) is also recommended; rationale | 3-5 |
|  | Item 6. Researcher characteristics and reflexivity: Researchers’ characteristics that may influence the research, including personal attributes, qualifications/experience, relationship with participants, assumptions, and/or presuppositions; potential or actual interaction between researchers’ characteristics and the research questions, approach, methods, results and/or transferability. | 4 |
| Context | Item 7. Context: Setting/site and salient contextual factors; rationale. | 4 |
| Sampling strategy | Item 8. Sampling strategy: How and why research participants, documents, or events were selected; criteria for deciding when no further sampling was necessary (e.g., sampling saturation); rationale. | 4 |
| Ethical issues pertaining to human subjects | Item 9. Ethical issues pertaining to human subjects: Documentation of approval by an appropriate ethics review board and participant consent, or explanation for lack thereof; other confidentiality and data security issues. | Title Page |
|  | Item 10. Data collection methods: Types of data collected; details of data collection procedures including (as appropriate) start and stop dates of data collection and analysis, iterative process, triangulation of sources/methods, and modification of procedures in response to evolving study findings; rationale. | 4 |
| Data collection instruments and technologies | Item 11. Data collection instruments and technologies: Description of instruments (e.g., interview guides, questionnaires) and devices (e.g., audio recorders) used for data collection; if/how the instrument(s) changed over the course of the study. | 4  Appendix 1 |
| Units of study | Item 12. Units of study: Number and relevant characteristics of participants, documents, or events included in the study; level of participation. | 6 |
| Data processing | Item 13. Data processing: Methods for processing data prior to and during analysis, including transcription, data entry, data management and security, verification of data integrity, data coding and anonymization / de-identification of excerpts. | 4 |
| Data analysis | Item 14. Data analysis: Process by which inferences, themes, etc. were identified and developed, including the researchers involved in data analysis; usually references a specific paradigm or approach; rationale. | 4-5 |
| Techniques to enhance trustworthiness | Item 15. Techniques to enhance trustworthiness: Techniques to enhance trustworthiness and credibility of data analysis,(e.g., member checking, triangulation, audit trail); rationale | 4 |
| Synthesis and interpretation | Item 16. Synthesis and interpretation: Main findings (e.g., interpretations, inferences, and themes); might include development of a theory or model, or integration with prior research or theory. | 6-16 |
| Links to empirical data | Item 17. Links to empirical data: Evidence (e.g., quotes, field notes, text excerpts, photographs) to substantiate analytic findings. | Table 3, page 13-16 |
|  | Item 18. Integration with prior work, implications, transferability, and contribution(s) to the field: Short summary of main findings, explanation of how findings and conclusions connect to, support, elaborate on, or challenge conclusions of earlier scholarship; discussion of scope of application/generalizability; identification of unique contribution(s) to scholarship in a discipline or field. | Table 2 page 9 |
| Limitations | Item 19. Limitations: Trustworthiness and limitations of findings | 17 |
| Conflicts of interest | Item 20. Conflicts of interest: Potential sources of influence or perceived influence on study conduct and conclusions; how these were managed. | Title Page |
| Funding | Item 21. Funding: Sources of funding and other support; role of funders in data collection, interpretation, and reporting. | Title Page |
